# Antibacterial Mechanism of Silkworm Seroins

**DOI:** 10.3390/polym12122985

**Published:** 2020-12-14

**Authors:** Hongtao Zhu, Xiaolu Zhang, Mengyao Lu, Haiqin Chen, Shiyi Chen, Jiaxuan Han, Yan Zhang, Ping Zhao, Zhaoming Dong

**Affiliations:** 1 State Key Laboratory of Silkworm Genome Biology, Southwest University, Chongqing 400716, China; edi_zhu@163.com (H.Z.); xiaolushg@126.com (X.Z.); lmy991217@126.com (M.L.); chq1245@126.com (H.C.); chenshiyi0804@163.com (S.C.); kingdihan6@163.com (J.H.); zhangy66@swu.edu.cn (Y.Z.); zhaop@swu.edu.cn (P.Z.); 2 Biological Science Research Center, Southwest University, Chongqing 400716, China; 3 Chongqing Engineering and Technology Research Center for Novel Silk Materials, Chongqing 400716, China

**Keywords:** seroin, silk protein, antibacterial protein, *Bombyx mori*

## Abstract

Seroin 1 and seroin 2 are abundant in silkworm cocoon silk and show strong antibacterial activities, and thus are thought to protect cocoon silk from damage by bacteria. In this study, we characterized the expression pattern of silkworm seroin 3, and found that seroin 3 is synthesized in the female ovary and secreted into egg to play its roles. After being infected, seroin 1, 2, and 3 were significantly up-regulated in the silkworm. We synthesized the full-length protein of seroin 1, 2, and 3 and their N/C-terminal domain (seroin-N/C), and compared the antimicrobial activities in vitro. All three seroins showed higher antibacterial activity against Gram-positive bacteria than against Gram-negative bacteria. Seroin 2 showed better antibacterial effect than seroin 1 and 3, whereas seroin 1/2/3-N was better than seroin 1/2/3-C. We found that seroin 2-C has stronger peptidoglycan binding ability than seroin 2-N per the ELISA test. The binding sites of seroin 2 with bacteria were blocked by peptidoglycan, which resulted in the loss of the antibacterial activity of seroin 2. Collectively, these findings suggest that seroin 1 and 2 play antibacterial roles in cocoon silk, whereas seroin 3 functions in the eggs. The three silkworm seroins have the same antibacterial mechanism, that is, binding to bacterial peptidoglycan by the C-terminal domain and inhibiting bacterial growth by the N-terminal domain.

## 1. Introduction

Silkworm (*Bombyx mori*) silk is a natural protein polymer with agricultural and economic significance [1]. As a model organism of lepidopteran insects, *B. mori* is the most well-known and earliest-used silk-secreting animal. *B. mori* silk and silk proteins have been extensively researched in-depth and are the most widely used [2,3,4,5,6]. Fibroins and sericins, as the main components of silk, are the macromolecular silk proteins. In addition, silk also contains many proteins with small size, including seroins and protease inhibitors, which are mixed with sericins. [7,8,9]. Seroins were first reported in the silk of *Galleria mellonella* and *B. mori*, named after the specific expression pattern in the middle silk gland (sericin) and the posterior silk gland (fibroin) [10,11]. Seroin 1 was found to be synthesized by the whole silk gland and secreted into both the fibroin and sericin layers. In contrast, seroin 2 is mainly found in the anterior silk gland and secreted into the outermost sericin layer. In addition to seroin 1 and 2, a new silkworm seroin was recently identified and named seroin 3 [12,13]. In 2019, Kucerova et al. divided the seroins into three subfamilies, Sn1, Sn2, and Sn3. Previously reported seroins 1 and 2 were classified as subfamily Sn1, seroin 3 was classified as subfamily Sn3, and Sn2 subfamily was newly identified, including three members in the silkworm: SN2a, Sn2b, and Sn2c [14]. Seroins were found to have two domains: a proline-rich N-terminal domain and a conserved C-terminal domain [13].

Singh et al. produced seroin 1 and 2 recombinant proteins using a prokaryotic expression system and evaluated their antibacterial activity in vitro. They found that seroin 2 efficiently inhibits growth of Gram-negative *Escherichia coli* and Gram-positive *Micrococcus luteus*, whereas seroin 1 only inhibits the growth of Gram-positive *M. luteus* [15]. Dong et al. reported that the specific distribution of seroin 2 in the surface of silk allows it to directly contact pathogens in the environment and to more efficiently play an antimicrobial role. Seroin 1 was thought to have dual roles in the silk: to prevent microbial invasion and to participate in silk fibroin complex formation as a molecular chaperone [13]. The function of seroin 3 has yet not been studied.

Although years of research have been conducted on the *B. mori* seroin proteins, many questions remain unanswered. For example, why do seroins have high abundance in the silk instead of antibacterial peptides? Whether seroin 3 has antibacterial activity and where seroin 3 functions is still unknown. What are the functions of seroin’s N-terminal and C-terminal domains? In this study, we revealed when and where seroin 3 works. Then, we compared the antibacterial activities among seroins 1, 2, and 3. To explain the bacteriostatic mechanism of seroin proteins, we divided seroins into two parts, the N-terminal domain and the C-terminal domain, and then evaluated their antibacterial activity and peptidoglycan binding ability in vitro. This article provides important clues to understanding the coexistence and coordination of protein polymer (fibroins, sericins, and chorions) and antimicrobial proteins (seroins).

## 2. Materials and Methods

### 2.1. Bioinformatics Analysis and Statistical Analysis

The nucleotide and amino acid sequences were downloaded from the silkworm genome database Silk DB (https://silkdb.bioinfotoolkits.net) and NCBI (https://www.ncbi.nlm.nih.gov). We analyzed the signal peptide, molecular weight, and isoelectric point of the amino acid sequences of silkworm seroin 1 (gi|19070653), seroin 2 (gi|19070655), and seroin 3 (gi|512931752) using SignalP (http://www.cbs.dtu.dk/services/SignalP-4.0/), ExPASy, BioEdit, and Clustal X [11,16,17]. To determine the statistical analysis of all the data in the experimental results, we used Student’s *t*-test.

### 2.2. Protein Truncation and Peptides Synthesis

According to previous reports, seroins 1, 2 and 3 were divided into two parts, N-terminal and C-terminal, the signal peptide was deleted, and the His tag was added to the front of the sequence to synthesize full-length and truncated peptides (GeneCreat Biotech, Wuhan, China) [13].

### 2.3. Sample Preparation

The *B. mori* strain Dazao (maintained in the Biological Science Research Center at the Southwest University of China) was reared with mulberry leaves at 25 °C and 75% ± 5% relative humidity. Different tissue samples were collected, including head, integument, fat body, silk gland, gonad, Malpighian tubule, midgut, and hemolymph, from day 5 of the fifth instar; head, integument, fat body, gonad, and hemolymph from day 5 of pupa; and head, wings, legs, thorax, abdominal epidermis, fat body, and gonad from day 1 of the moth.

*Escherichia coli* and *Staphylococcus aureus* (BeiNa Culture Collection, Beijing, China) were cultured on Luria-Bertani (LB) medium for 12 h at 37 °C. After removing the medium, wash the cells with sterilized phosphate buffered saline (PBS, pH 7.2) and diluted to 1 × 10^5^ conidia/mL. Each silkworm larva on day 3 of the fifth instar was injected with bacteria dilution liquid (10 μL), and sterile PBS was injected into larvae to set the control group. The hemolymph and fat bodies of 20 silkworms were collected from each group every hour from 0 to 8 h after injection. *Saccharomyces cerevisiae* and *Candida albicans* (BeiNa Culture Collection, Beijing, China) were cultured on potato dextrose agar (PDA) medium for 15 days at 30 °C. Fungal conidia were harvested from cultures of the aforementioned species and suspended in distilled water containing 0.05% (*v*/*v*) Tween-80 at a concentration of 1 × 10^5^ conidia/mL after being filtered through sterilized absorbent cotton. Each silkworm larva on day 3 of the fifth instar injected with fungal spore dilution liquid (10 μL), and sterile PBS solution was injected into larvae to set the control group. The hemolymph and fat bodies of 20 silkworms were collected from each group 0, 6, 12, 18, and 24 h after injection. All the hemolymph was collected in a centrifuge tube containing phenylthiourea and then centrifuged at 4 °C for 10 min at 1000 ×*g* to remove blood serum.

### 2.4. Real-Time Quantitative Reverse Transcription PCR

Total RNAs were extracted from samples of tissues each period or after microbial infection by using an RNApure total RNA rapid extraction kit (Magen, Guangzhou, China). Reverse transcription into cDNA was performed using M-MLV reverse transcriptase (Invitrogen, Carlsbad, CA, USA). Real-time quantitative PCR (qPCR) was performed in a qTOWER2.2 qPCR machine (Analytikjena Biometra, Jena, Germany). The primers were designed based on the previously published gene sequences (Table 1). Each amplification reaction was performed in a total volume of 20 μL containing 150 ng cDNA (2 μL), 10 μL SYBR Premix Ex Taq II (Takara, Shiga, Japan), and 0.4 μM primers, under the following conditions: an initial denaturation at 95 °C for 60 s, followed by 40 cycles of denaturation at 95 °C for 20 s, annealing at 60 °C for 60 s, and extension at 72 °C for 35 s. Data results were further verified by the 2^−ΔΔ*CT*^ method. Fold changes in *seroin 1*, *seroin 2*, and *seroin 3* expressions upon bacterial and saline infection were compared with the control (PBS) group.

### 2.5. Immunofluorescence Analysis

Silkworm eggs were embedded in optimal cutting temperature compound (OTC) agent (Sakura, Shiga, Japan). Frozen slices of eggs 8 μm thick were obtained using a Leica CM1950 microtome (Leica, Wentzlar, Germany), which were washed three times (each for 10 min) with PBS. Then, the slices were treated with PBS containing 1% bovine serum albumin (BSA; Sigma-Aldrich, St. Louis, MO, USA) and 10% normal goat serum for 1 h at 25 °C. After subsequent incubation with an anti-seroin 3 rabbit antibody (1:300 in PBS containing 1% BSA) (Zeheng Biotech, Chongqing, China) for 2 h at 25 °C, the slices were washed three times (each for 10 min) with PBST (PBS containing 0.1% Tween), and thereafter incubated for 1 h with Cy3-labeled goat anti-rabbit IgG (H + L) (Beyotime, Shanghai, China) at a dilution of 1:500 in PBS containing 1% BSA followed by three 10 min washes with PBST. Fluorescence in the tissue samples was observed under an LSM 880 confocal microscope (Zeiss, Jena, Germany).

### 2.6. Western Blot Analysis

The tissues proteins were extracted in 9 M LiSCN by homogenization and centrifuged at 16,000× *g* for 20 min at room temperature. The supernatants (10 mg protein) were separated by 12.5% SDS-PAGE, and then transferred to polyvinylidene fluoride (PVDF) membranes for Western blot analysis. After treatment with 5% nonfat dry milk in 50 mM Tris-HCl (pH 7.5), 500 mM NaCl, and 0.1% Tween 20 (TBST) for 1 h, the membranes were incubated with the seroin 3 polyclonal rabbit anti-seroins3 (1:20,000 and 1:10,000, respectively) (Zeheng Biotech, Chongqing, China). After being washed five times with TBST for 5 min to remove any excessive antibody, the membranes were incubated with corresponding secondary antibody for 1 h. The corresponding secondary antibody, horseradish peroxidase (HRP) labeled goat anti-rabbit antibody (1:40,000, Beyotime, Shanghai, China) was added for 1 h, followed by washing three times with TBST for 10 min. Signals were detected with the Amersham ECL™ Advance Western Blotting Detection Kit (GE Healthcare, Chicago, IL, USA) and scanned using the Chemi-scope 3400 mini-instrument (Clinx Science, Shanghai, China).

### 2.7. Bacteriostatic Analysis

For bacteria, growth inhibition assays were performed in flat-bottom 96-well plates (Corning, Corning, NY, USA). Antimicrobial activity of the seroins was estimated against Gram-negative *E. coli* and Gram-positive *S. aureus*. Each well was filled with 200 μL LB medium containing bacteria, and cultured in LB medium until the absorbance of at 600 nm reached 0.2. Fungi growth inhibition assays were performed in flat-bottom 96-well plates. Each well was filled with 200 μL potato liquid medium containing fungal conidia at a final concentration of 1 × 10^4^ conidia/mL. The culture medium contained chloramphenicol to prevent bacterial infection.

We added 25 μg of peptides of different seroin proteins to each well of the 96-well plates. The microplates were incubated at 37 °C for bacteria and 30 °C for fungi. The growth was observed by monitoring the absorbance at 600 nm (optical density, OD_600_) after culturing for 0, 1, 2, 3, 4, 5, 6, 7 and 8 h for bacteria and 0, 12, 24, 36, 48, 60 and 72 h for fungi. BSA and ethylenediaminetetraacetic acid (EDTA) were used as negative and positive controls, respectively, whereas PBS was used as the blank control. Synthesized peptides and N-terminal and C-terminal of seroins 1, 2, and 3 were used as the experimental groups. All data were normalized with the PBS groups as the standard. The growth rate was calculated as:

$$Growth\;rate\;at\;n\;h=\frac{OD_{600}\;at\;n\;h\;-\;OD_{600}\;at\;0\;h}{OD_{600}\;at\;0\;h}$$

### 2.8. Peptidoglycan Binding and Competition Assay

We used ELISA kits (Invitrogen, Carlsbad, CA, USA) to detect the peptidoglycan (PGN) binding ability of seroin proteins. We added 10 µg *S. aureus* PGN dissolved in blocking buffer (Yuanye Bio-Technology, Shanghai, China) to each well of the 96-well ELISA microplates for cross-linking. We aspirated the uncoated polysaccharide from the microplates, added BSA blocking buffer, and blocked for 1 h. Different concentrations of seroin synthetic peptides were added to the microplates and then incubated for 3 h. Then, the His-tag antibody was added, and the same volume of protein non-immune serum was added to the negative control group and incubated for 1 h. We added HRP-labeled secondary antibody to the microplates, which were incubate for 1 h, then we added the tetramethylbenzidine (TMB) color-developing solution (Invitrogen, Carlsbad, CA, USA). After incubating for 30 min in a dark place, we added an equal volume of 0.2 M H_2_SO_4_ to each well to stop the color developing reaction. We used the spectrophotometer (Gene, Shanghai, China) to read and recorded the absorbance of OD_450_. The calculation formula is:

P (sample)N (negative)=OD450 of experimental group−OD450 of blank group OD450 of control group−OD450 of blank group

When *P*/*N* > 2.1, protein is considered to be bound to peptidoglycan [18,19,20].

For the peptidoglycan competition assay, 25 μg peptides was added to 50 μL 4 μg/μL PGN solution and 50 μL PBS solution as the experimental group and the control group, respectively, and incubated for 1 h. We added the incubated solution to a 96-well plate, and added an equal volume of PBS solution as a blank group. We added 150 μL of *S. aureus* bacterial solution (OD_600_ = 0.2) to each well of the 96-well plate, which was incubated at 37 °C; we recorded the absorbance of OD_600_ after culturing for 0, 1, 2, 3, 4, 5, 6, 7, and 8 h. All data were normalized with the PBS group as the standard.

## 3. Results

### 3.1. Bioinformatics Analysis of Seroin Proteins

**Figure 1:**
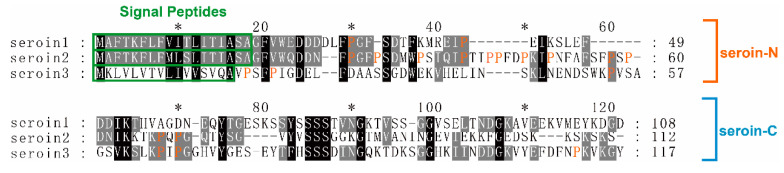
Multiple sequence alignment of 3 silkworm seroin proteins. The proteins sequences of seroin 1 (gi|19070653), seroin 2 (gi|19070655), and seroin 3 (gi|512931752) were downloaded from NCBI. The absolutely conserved residues are marked as black the relative conserved residues are marked as grey. The signal peptides are boxed and the proline is highlighted.

Using SignalP, ExPASy, and BioEdit to analyze the amino acid sequence of seroins 1, 2, and 3, we found that all three silkworm seroin proteins predicted signal peptides, and their molecular weight without signal peptides are 9.8, 10.3, and 11.0 kDa, respectively. The predicted isoelectric point (pI) were 4.13, 8.94, and 5.22, respectively. The analysis results are shown in the Table 2. By comparing the sequence of the three seroin proteins, we found that seroins 1 and 2 have relative higher sequence homology of the three seroins (Figure 1).

### 3.2. Expression Pattern of Seroin 3

**Figure 2:**
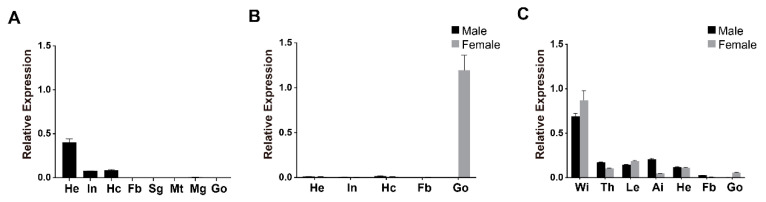
Analysis of the transcriptional expression of *seroin 3*. The mRNA expression of *seroin 3* was detected in different tissues from day 5 of the fifth instar larvae (**A**), day 5 of the pupae (**B**), and day 1 of the moth (**C**). He: head; In: integument; Hc: hemocytes; Fb: fat body; Sg: silk gland; Mt: Malpighian tubule; Mg: midgut; Go: gonad; Wi: wing; Th: thorax; Le: leg; Ai: abdomen integument. The y-axis indicates the relative expression level of *seroin 3* mRNA transcripts. Error bars indicate the standard error of the mean (*n* = 3).

Since previous studies have revealed the expression pattern of seroins 1 and 2, we focused on the temporal and spatial expression profile of seroin 3. The transcriptional expression of *seroin 3* was detected by qPCR with *seroin-3*-specific qPCR primers and using cDNA from different tissues of silkworm larvae, pupae, and adults as templates. In the larvae of the last instar, *seroin 3* was found to be expressed highly in the head, but weakly in the epidermis and hemocyte (Figure 2A). In the pupal stage, *seroin 3* was only expressed in the ovary. In the adult stage, it was highly expressed in the wings, an in other tissues such as thorax, leg, and abdomen integument (Figure 2).

**Figure 3:**
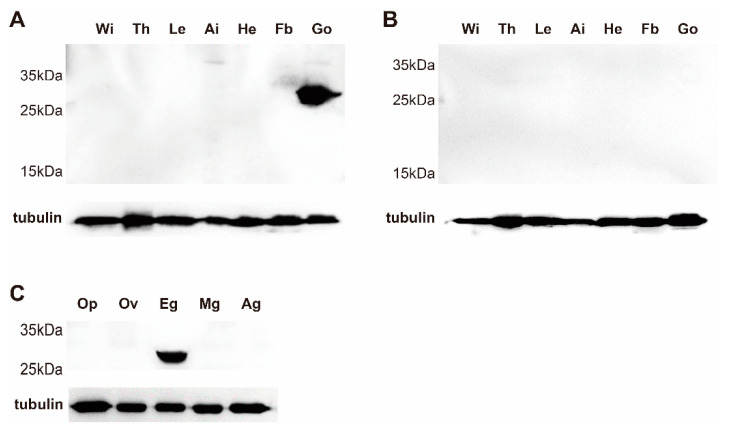
Western blot detection of seroin 3 in different tissues from female (**A**) and male moths (**B**) and different parts of ovary from a female moth (**C**). Wi: wing; Th: thorax; Le: leg; Ai: abdomen integument; He: head; Fb: Fat body; Go: gonad; Op: ovipositor; Ov: ovipositor; Eg: egg; Mg: mucous gland; Ag: alluring gland.

**Figure 4:**
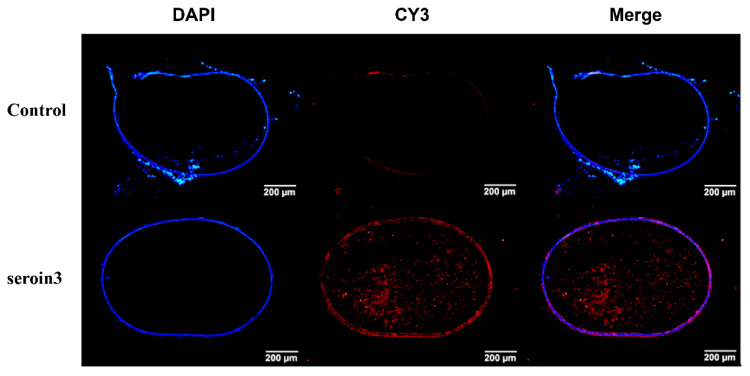
Immunofluorescence analysis of seroin 3 in eggs. Slides were incubated with seroin 3 antibodies followed by a secondary antibody labeled with cy3 (red) and counterstained with 4′,6-diamidino-2-phenylindole (DAPI, blue). Control experiments were also performed using pre-immune serum.

To detect the protein distribution of seroin 3, tissue proteins were extracted from larval, pupal, and adult stages. The abundance of seroin 3 was detected by Western blot using the polyclonal antibody of seroin 3. Seroin 3 was only be detected in the adult stage, but not in the larval and pupal stages. In the moth, seroin 3 was specifically expressed in the ovary of the female, but not in other tissues. Further detection revealed that seroin 3 was only in the eggs but not in other parts of the ovary (Figure 3). The frozen sections of eggs and immunofluorescence experiments verified that seroin 3 protein is abundantly expressed in the eggshell and inside the eggs (Figure 4).

### 3.3. Upregulation of the Seroins in Response to Microorganism Infection

**Figure 5:**
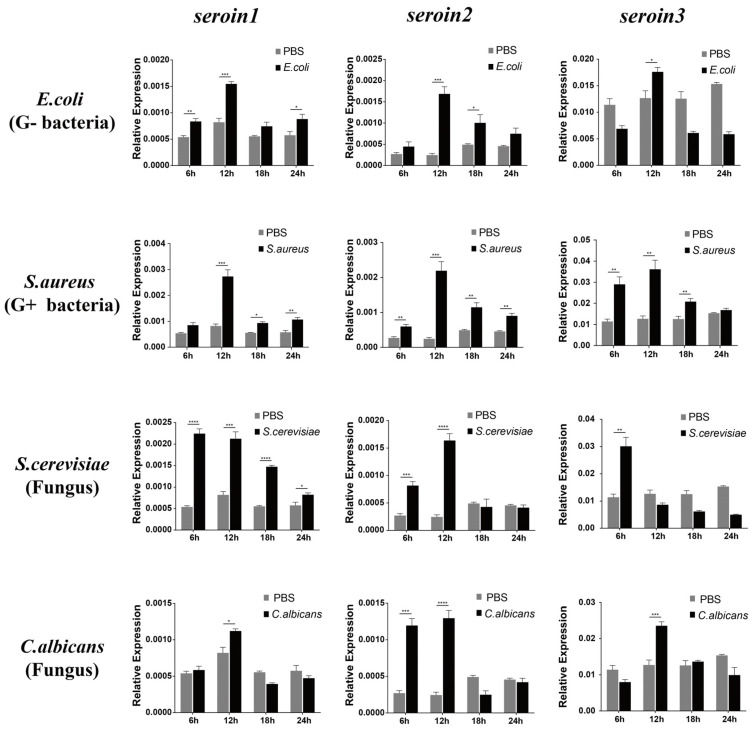
The mRNA expression pattern of *seroin 1, 2,* and *3* in the hemocytes of *B. mori* larvae infected with different microorganisms. The y-axis indicates the relative expression levels of *seroin 1/2/3* mRNA transcripts. Student’s *t*-test was used to evaluate statistical significance. Vertical bars represent the mean ± standard error (*n* = 3). ** *p* < 0.001 and * *p* < 0.01 versus control. Error bars indicate the standard error of the mean (*n* = 3).

The expressions of *seroin 1*, *seroin 2* and *seroin 3* in silkworm hemocytes were detected after being infected by *E. coli* (Gram-negative bacteria), *S. aureus* (Gram-positive bacteria), *S. cerevisiae* (fungus), and *C. albicans* (fungus). The results showed that all three seroin genes were significantly upregulated at different time points especially at the sixth hour after being infected with Gram-negative bacteria, Gram-positive bacteria, and fungi. Among the three seroins, the upregulation of *seroin 2* was stronger than that of *seroin 1* and *seroin 3* (Figure 5).

### 3.4. Antimicrobial Activity of the Seroin Proteins In Vitro

**Figure 6:**
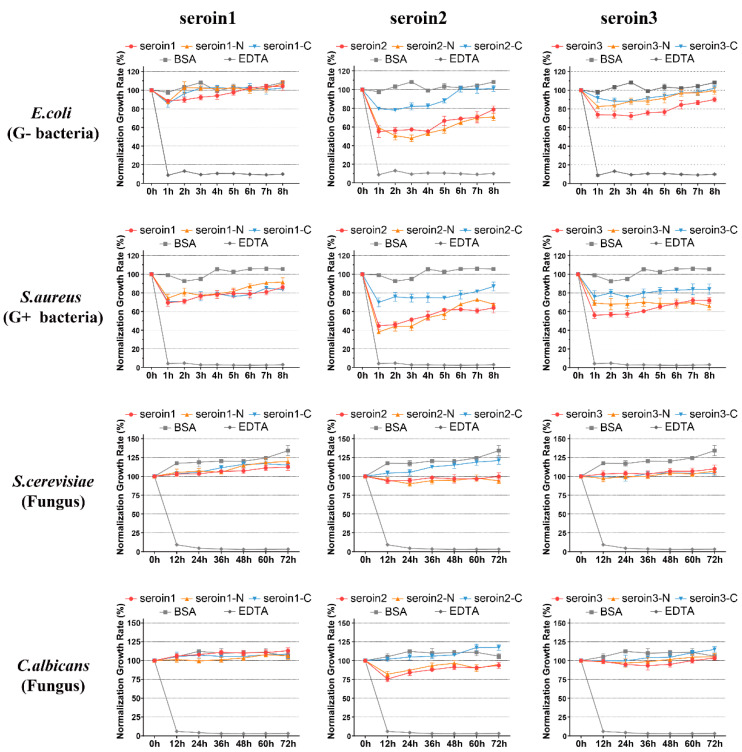
Bacteriostatic activity of seroins against *E. coli*, *S. aureus*, *S. cerevisiae*, and *C. albicans*. The full-length proteins of seroins 1, 2, and 3, and seroins 1-, 2-, and 3-derived N-terminal and C-terminal peptides were used for bacteriostatic experiment. Bovine albumin (BSA) was used as the negative control, and ethylene diamine tetra acetic acid (EDTA) as the positive control. Vertical bars represent the mean ± standard error (*n* = 3).

The antibacterial activity of seroin 1 and seroin 2 have been previously evaluated, but the antibacterial activity of seroin 3 was unknown. Since seroin proteins have two domains, the Pro-rich N-terminal domain (seroin-N) and the conserved C-terminal domain (seroin-C), we synthesized the full-length proteins, the N-terminal peptides, and the C-terminal peptides of seroins 1, 2, and 3, and then tested and compared their antimicrobial activities in vitro (Figure 6). 

First, we studied the bacteriostatic activity of seroin proteins on bacteria. Seroin 1, seroin 1-N, and seroin 1-C have almost no bacteriostatic activity on the growth of Gram-negative *E. coli*, but have an obvious bacteriostatic effect on Gram-positive *S. aureus*. Seroin 2 showed better antibacterial activity against *E. coli* than *S. aureus*, whereas seroin 2 and seroin 2-N showed obviously better antibacterial activity than seroin 2-C. Seroin 3 has relative stronger bacteriostatic activity against *S. aureus* than *E. coli*, whereas seroin 3 and seroin 3-N have better antibacterial activity than seroin 3-C. Collectively, we found seroins have stronger antibacterial activity against *S. aureus* than *E. coli*, seroin 2 is stronger than seroins 1 and 3, and seroin-N is stronger than seroin-C.

Previous studies tested the bacteriostatic of seroin proteins on bacteria and viruses, but did not clarify whether they can inhibit fungi. Therefore, we chose two fungi, *S. cerevisiae* and *C. albicans*, for antifungal experiments. The results showed that seroin proteins have no or weak antifungal activity. In contrast, seroin 2 and seroin 2-N have better antifungal activity than seroin 1, seroin 1-N, seroin 3, and seroin 3-N.

### 3.5. Peptidoglycan Binding and Competition Assay

**Figure 7:**
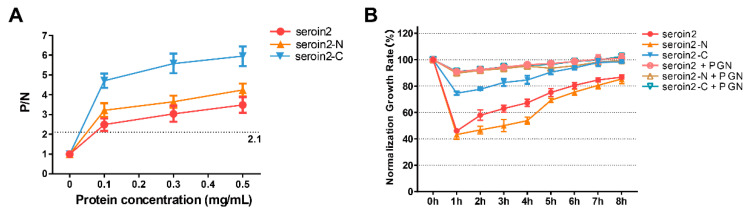
Peptidoglycan (PGN) binding and competition assay. (**A**) ELISA analysis of binding profile between seroin 2/2-N/2-C and PGN. The negative control is unimmunized serum, and empty wells were used as blanks. Samples with *P (sample)*/*N (negative)* > 2.1 were considered positive. Student’s *t*-test was used to evaluate statistical significance. (**B**) The bacteriostatic activity of seroin 2/2-N/2-C against *S. aureus*. Seroin 2, seroin 2-N, and seroin 2-C were incubated with or without peptidoglycan and then with *S. aureus*. Error bars indicate the standard error of the mean (*n* = 3).

Peptidoglycan (PGN) can block the binding sites of seroins with bacteria and thus affect the antibacterial activity. Thus, we selected seroin 2, the strongest antibacterial seroin, and its truncated peptides to test the PGN-binding activity (Figure 7). The results showed that seroin 2, seroin 2-N, and seroin 2-C bound more peptidoglycan as the protein concentration increased, and seroin 2-C had stronger PGN-binding ability than seroin 2-N. After being pre-incubated with PGN, seroin 2, seroin 2-N, and seroin 2-C lost their antibacterial activity.

## 4. Discussion

*B. mori* seroin 1 and seroin 2 were found to have antibacterial and antiviral activity in a previous study [15]. Our recent research identified a new seroin in the silkworm, which was named seroin 3. We found it is not expressed in the silk gland. We also found that seroins have two domains: the proline-rich N-terminal domain and conserved C-terminal domain [13]. To reveal the antimicrobial mechanism of seroins, we investigated the expression profile and antibacterial activity of seroin 3. We found that seroin 1-, 2-, and 3-derived N-terminal and C-terminal peptides play different roles in the antibacterial process.

We analyzed the antibacterial activities of the seroins 1, 2, and 3 against a variety of microorganisms, including Gram-negative bacteria (*E. coli)*, Gram-positive bacteria (*S. aureus)*, and two fungi (*S. cerevisiae* and *C. albicans*). We found the antibacterial activity of seroins against Gram-positive bacteria is better than against Gram-negative bacteria, consistent with a previously report [15]. The antifungal activity of seroins is reported for the first time, although it is weaker than the antibacterial activity. Among three seroins, seroin 2 has the strongest antibacterial activity, followed by seroin 3 and then seroin 1. Interestingly, we found that seroin 2 has higher-fold upregulation after microbial induction than seroins 1 and 3. Thus, we speculate that seroin 2 is the major effector gene that eliminates microbial infection among three seroins as seroin 2 has a broader antimicrobial spectrum, stronger antimicrobial activity, and higher microbe-induced expression than seroins 1 and seroin. The status of seroin 2 compared to other seroins may be similar to the status of cecB6, cecD and mor to other antimicrobial peptides [21]. However, seroin 1 has poor antibacterial activity, but its content in the silk is higher than seroin 2. We think the main function of seroin 1 may shift from antibacterial to participating in the synthesis and assembly of silk proteins. Previous studies reported that the expression pattern of seroin 3 is different from that of seroin 1 and seroin 2, and it is not expressed in the silk gland [12,13]. We found that seroin 3 is synthesized in the pupal ovaries and secreted into eggs in the moth stage. These results indicated that seroin 3 functions in a different location than seroin 1 and seroin 2: not in the silk but in the eggs.

Since seroin protein has two domains, the proline-rich N-terminal domain and the conserved C-terminal domain [13], we speculate that they play different biological roles. The antibacterial activity of seroin 1/2/3-N was obviously stronger than seroin 1/2/3-C, and the antibacterial activity of seroin 2-N was obviously stronger than that of seroin 1-N and seroin 3-N. The reason for the above results might be the seroin-N has high proportion of proline (6.45–23.8%) than seroin-C (0–5%), and seroin 2-N (23.8%) has a higher proline content than seroin 1-N (6.45%) and seroin 3-N (7.32%) [22,23,24,25,26,27,28].

Due to seroins having better antibacterial activity against Gram-positive bacteria than Gram-negative bacteria, we speculate that seroins may have better binding ability with Gram-positive bacteria than Gram-negative bacteria. The cell walls of Gram-positive bacteria are composed predominantly of peptidoglycan, whereas the Gram-negative bacteria cell wall has a thin peptidoglycan layer. Our results suggested that the C-terminal domain of seroin has better peptidoglycan binding ability than the N-terminal domain. Collectively, the findings revealed the antibacterial mechanism of seroins, that is, binding to the bacterial peptidoglycan by the C-terminal domain and inhibiting bacterial growth by the N-terminal domain.

Although we observed that the three seroin proteins all have antibacterial activity, compared with some previously reported antimicrobial peptides from bacteria, human, or other sources [29,30,31], the seroin proteins showed relatively weaker antibacterial activity. The reason why the silkworm choses to synthesize seroins in silk and eggs rather than antimicrobial peptides as a defense function remains unknown [21]. Future research could perform an in-depth analysis of this aspect in order to answer this question.

## Figures and Tables

**Table 1:** Primer sequences.

Primer	Sequences
seroin 1-qF	GTCGCGGGAGATAACGAG
seroin 1-qR	CCGTTGACCGTGGATGAG
seroin 2-qF	GCTGGCTTTGTTTGGCAGGACG
seroin 2-qR	CCTTCCCTCCATTGCTGCTCAC
seroin 3-qF	GTTTGATGCTGCCTCTT
seroin 3-qR	TTCTGTCCGTTGATGTCT

**Table 2:** Analysis of biological information of seroin proteins. AA: amino acids, MW: molecular weight, pI: isoelectric point.

Protein	Length (aa)	Signal (aa)	Proline Content	MW (kDa)	pI
seroin 1	108	18	2 aa, 1.85%	9.8	4.13
seroin 1-N	31	/	2 aa, 6.45%	3.64	3.76
seroin 1-C	59	/	/	6.22	4.42
seroin 2	112	18	12 aa, 10.7%	10.3	8.94
seroin 2-N	42	/	10 aa, 23.8%	4.78	3.77
seroin 2-C	52	/	2 aa, 3.85%	5.52	9.7
seroin 3	117	16	6 aa, 5.13%	11.0	5.22
seroin 3-N	41	/	3 aa, 7.32%	4.53	4.28
seroin 3-C	60	/	3 aa, 5%	6.56	7.02
